# Associations between alcohol use disorders and adherence to antiretroviral treatment and quality of life amongst people living with HIV/AIDS

**DOI:** 10.1186/1471-2458-14-27

**Published:** 2014-01-10

**Authors:** Bach Xuan Tran, Long Thanh Nguyen, Cuong Duy Do, Quyen Le Nguyen, Rachel Marie Maher

**Affiliations:** 1 Institute for Preventive Medicine and Public Health, Hanoi Medical University, Hanoi, Vietnam; 2 Authority of HIV/AIDS Control, Ministry of Health, Hanoi, Vietnam; 3 Department of Infectious Diseases, Bach Mai Hospital, Hanoi, Vietnam; 4 School of Medicine and Pharmacy, Vietnam National University, Hanoi, Vietnam

**Keywords:** Alcohol use disorders, HIV/AIDS, Antiretroviral treatment, WHOQOL-HIV, Adherence, Vietnam

## Abstract

**Background:**

We examined the association of alcohol use disorders (AUD) with adherence to and health-related quality of life (HRQOL) outcomes of antiretroviral treatment (ART) for HIV/AIDS patients.

**Methods:**

A cross-sectional multi-site survey was conducted in 468 drug users and 648 non-drug users (age: 35.4 ± 7.0 years; 63.8% male) in 3 epicentres of Vietnam. AUD, ART adherence, and HRQOL were measured using the Alcohol Use Disorders Identification Test - Consumption (AUDIT-C), the self-reported Visual Analogue Scale (VAS), and the World Health Organization Quality of Life instrument (WHOQOL-HIV BREF).

**Results:**

35.0% of drug users were hazardous drinkers, compared to 25.9% of non-drug users. 22.3% of drug users engaged in binge drinking, and 25.9% reported suboptimal ART adherence. Adjusting for propensity scores of AUD, patients who had either at-risk or binge drinking behaviour were about twice as likely to be treatment non-adherent as those who did not have AUD. Hazardous drinkers reported small to medium decrements in the Performance, Physical, Social, Spirituality, and Environment quality of life domains. Binge drinkers had a slightly higher score in Social dimension.

**Conclusion:**

AUD is prevalent and negatively affecting adherence to and HRQOL outcomes of ART services in injection-driven HIV epidemics. Screening and intervention are recommended for AUD, especially during the stable periods of ART. Other social and psychological interventions might also enhance patients’ responses to and outcomes of ART in Vietnam.

## Background

In many Asian populations, hazardous alcohol use is found to be associated with the spread of HIV infection and substantial unfavourable outcomes of HIV/AIDS treatment [1-4]. At-risk drinkers are more likely to engage in unprotected sex, which contributes to the transmission of HIV and other sexually transmitted infections [1]. Among HIV/AIDS patients, hazardous drinkers adhered less to antiretroviral treatment (ART) than other patient groups, resulting in poorer immunological and virological treatment outcomes [5]. Alcohol use is also found to be associated with lipodystrophy and exacerbate antiretroviral therapy - induced neuropathic pain in patients with HIV/AIDS [6,7]. Moreover, it has a direct association with depression and HIV disease progression [8]. Interventions for individuals with substance abuse - including alcohol - are therefore necessary measures to control the spread and reduce the impact of HIV/AIDS.

The HIV epidemics in Asia are largely driven by drug injection, and more than half of all people living with HIV/AIDS in these countries are injection drug users [4]. Treatment of opiate drug use during ART has been implemented in some settings, such as the integration of methadone maintenance with ART services [9,10]. However, while a high prevalence of alcohol use disorders (AUD) has also been observed among drug users, its negative impact on the outcomes of ART is not fully recognized [11-16]. This lack of knowledge may have a couple of possible explanations. First, alcohol is a legal commodity which is culturally accepted in many Asian cultures [11]. In addition, few studies in Asia have quantified the impact of AUD on HIV/AIDS treatment outcomes, and empirical evidence of large injection-driven HIV epidemics is still limited.

Vietnam has a concentrated HIV epidemic, which emerged initially in drug using populations. It is estimated that 320,000 people have contracted HIV, 70% of which are drug users [17]. Antiretroviral treatment services have been rapidly scaled up in the country since 2006, and covered 60% of patients with HIV who were in need of treatment by 2012 [18]. Previous works have shown various factors that influenced adherence to and outcomes of antiretroviral treatment in the Vietnamese settings [18-23]. This included, for instance, avoidance of HIV testing, deferred antiretroviral treatment, heroin use, lack of social and familial support, stigma and discrimination. Although one third of HIV/AIDS patients are hazardous drinkers, the magnitude of AUD’s impacts on HIV treatment outcomes have not been determined, and not any intervention of alcohol use among patients with HIV/AIDS has been implemented [11]. In this study we sought to examine the association of AUD on antiretroviral treatment adherence and health-related quality of life (HRQOL) of HIV/AIDS patients receiving treatment from multiple ART clinics in three epicentres of Vietnam. The study provides a baseline for evaluating effectiveness of potential intervention strategies to reduce alcohol consumption among HIV/AIDS patients.

## Methods

### Study design and participant recruitment

This study was a part of the 2012 HIV Services Users Survey, which was conducted in seven clinics in three epicentres of Vietnam: Ha Noi, Hai Phong, and Ho Chi Minh City. The survey included inpatients and outpatients who were attending ART clinics in three district health centres, three provincial hospitals, and one central hospital. A detailed description of survey design and sampling has been presented elsewhere [11,24,25]. In short, we purposively selected facilities based on the following criteria: 1) the sample included central-, provincial- and district-level hospitals or health centres 2) they have been providing ART services, and 3) a sufficient number of HIV/AIDS patients attend each clinic. All HIV-positive inpatients and outpatients who were registering for care or taking ART at selected hospitals were eligible for the study. Since HIV-related information is confidential, it was not feasible to develop a sample frame. Therefore, we selected patients conveniently, including those who were present at the clinics during the study period, and who gave informed consent to participate in the study, until reaching at least 100 patients per site and 200 patients per clinic at the national level. A total of 1016 patients were selected, including 468 drug users and 548 non-drug users.

### Measures and instrument

Patients were interviewed using a structured questionnaire about their socioeconomic, clinical and behavioral characteristics. *Alcohol use consumption* was assessed using the *Alcohol Use Disorders Identification Test - Consumption* (AUDIT-C). It is a brief version of the 10-question AUDIT instrument, which consists of 3 questions: 1) How often do you have a dink containing alcohol?; 2) How many standard drinks containing alcohol do you have on a typical day?; and 3) How often do you have six or more drinks on one occasion? [26,27]. The AUDIT-C score ranged from 0-12, where 4 or more in men and 3 or more in women are considered active AUD or at-risk drinking. The third question, AUDIT-3, relates to binge drinking and is defined as positive if it receives any positive response [27]. *Antiretroviral treatment adherence* was self-reported over the past 30 days using a visual analogue scale (VAS) [28]. The VAS score ranged at [0; 100] where the threshold for optimal adherence was defined at 95% and above.

Patients were asked to complete a questionnaire about their HRQOL using *the World Health Organization Quality of Life - HIV Brief Instrument* (WHOQOL-HIV BREF). Those patients who were severely ill and who experienced any difficulty in completing the form were interviewed by study administrators. The WHOQOL-HIV BREF is a multidimensional profile which includes 31 items covering 6 domains and 2 other general items (Overall HRQOL and General Health) [29,30]. The respondents answered each question using a 5-item Likert scale. Average domain scores were multiplied by four to convert domain scores to the range of [4,20], making it comparable with scores derived from the WHOQOL-100. Development of the Vietnamese version and psychometric properties of WHOQOL-HIV BREF have been presented elsewhere [31,32]. In factor analysis, the items were re-classified into 6 modified domains, including: Performance (10 items), Physical (4 items), Morbidity (5 items), Social (4 items), Spirituality (3 items), and Environment (3 items).

### Statistical analysis

Impact of AUD on ART adherence and HRQOL outcomes were examined in multivariate regression models. Since the number of participants and their observed characteristics might be disproportionate between those patients with and without AUD, estimability of the models had the potential to be biased. To compensate for this, we used p*ropensity score* to reduce the pre-existing differences to a single dimension [33]. A propensity score is defined as the conditional probability of belonging to the AUD group given a vector of observed covariates which summarizes information across potential confounders [34]. Propensity scores of AUD (at-risk drinking and binge drinking) were estimated using logistic regression with predictors including socioeconomic status and HIV-related characteristics of respondents. Co-linearity was examined using the variance inflation factors. A stepwise forward model selection was applied, where variables were included based on the log-likelihood ratio test. We adopted a p-value <0.1, and excluded variables at p-values >0.2. The equations are expressed as follows:

$$\mathrm{LOGIT}\left[P\left(\mathrm{AUD}|\mathrm{SES},\mathrm{HIV}\right)\right]=\alpha +\sum_{i}\beta_{1i}\mathrm{SE}S_{i}+\sum_{i}\beta_{2i}\mathrm{HI}V_{i}$$

*Where:* SES and HIV represent socio-demographic and HIV-related characteristics of respondents.

*SES*_*i*_ included: sex, age (continuous), educational attainment (high school and above, others), marital status (single, live with spouse or partner, widow(er)/divorced/separated), employment (unemployed, stable jobs, unstable jobs), religion (Buddhism and others), income per capita (five quintiles).

*HIV*_*i*_ included length of time living with HIV, HIV stage, length of ART.

Propensity score is calculated as follows:

$$\mathrm{PROPENSITY}\;=\mathrm{Predict}\left[P\left(\mathrm{AUD}|\mathrm{SES},\mathrm{HIV}\right)\right]\;$$

*Propensity score - adjusted linear and logistic regression analysis* were used to determine the associations of at-risk and binge drinking with ART non-adherence and HRQOL.

Since WHOQOL-HIV BREF domain scores raged at [4,20], they actually were left and right censored. Censoring from above and below the WHOQOL-HIV BREF domain scores did not allow us to measure exactly the values which were higher or lower than the range thresholds. Therefore, in multivariate linear regression, we employed censored regression models or Tobit models to estimate linear relationships between AUD and HRQOL [35]. Differences in HRQOL scores between patients with and without AUD were then quantified into Cohen’s effect size, which is defined as the magnitude of differences divided by standard deviations of the sample measurements. Since drug use is a potential confounder of the association between AUD and ART adherence and outcomes, we stratified this analysis by history of drug use.

## Results

### Characteristics of participants

The sample population studied was 63.8% men and 36.2% women, who had a mean age was 35.4 (SD = 7.0). 45% had high school education and above, 64% lived with their spouses or partners, and 20.4% had stable jobs. A large proportion of patients in the sample had a history of drug use (46.1%), and 87% of them actively used drugs at the time of the study. The mean duration of HIV infection was 5.7 years (SD = 3.7 years) and 88.8% of patients had been taking ART for an average period of 3.0 years (SD = 2.1 years). The distribution of patients by ART duration period was as follows: 1^st^ year (19.3%), 2^nd^ year (14.2%), 2-4 years (26.6%), and 4-7 years (28.7%). 31% of patients had CD4 count less than 200 cells/μl, and 62.2% had less than 350 cells/μl.

### Alcohol consumption, ART adherence and HRQOL profile

**Figure 1:**
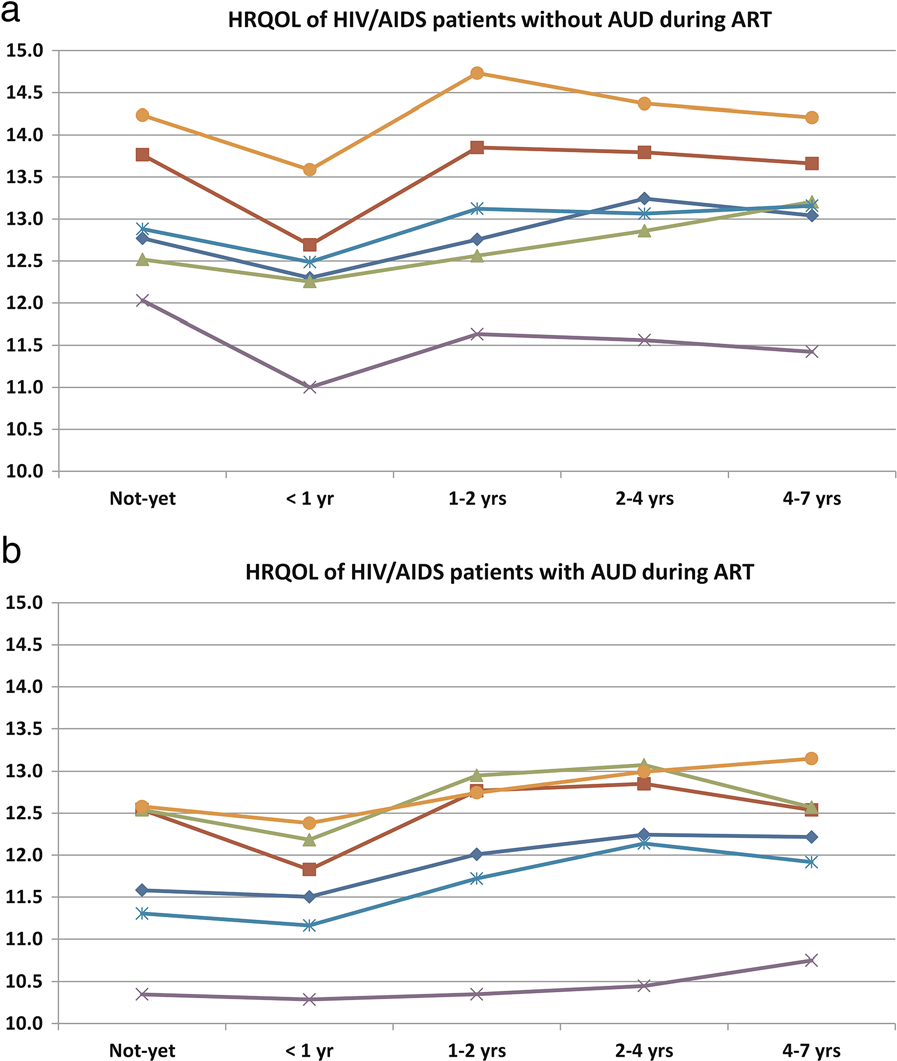
**HRQOL of HIV/AIDS patients at different periods of ART. a**. HRQOL of HIV/AIDS patients without AUD during ART. **b**. HRQOL of HIV/AIDS patients with AUD during ART.

Of the 1016 respondents, 30.1% were at-risk drinkers (35.0% among drug users, and 25.9% among non-drug users), 22.3% exhibited binge drinking with six or more drinks on one occasion, 25.9% patients reported non-adherence to ART. As indicated in Table 1, the percentage of at-risk drinking was higher in patients who were not yet on ART (40.4%) or who were on their 1^st^ year of ART (35.7%) than in other patients; meanwhile, there was no significant difference in the percentage of binge drinking across ART periods. The percentage of non-adherence to ART was higher in patients with AUD compared to those without AUD in the periods of 1-2 years and 4-7 years ART. The average HRQOL domain scores for all 1016 respondents was 12.6 (SD = 2.3) in Performance, 13.2 (SD = 3.1) in Physical, 12.7 (SD = 3.5) in Morbidity, 11.2 (SD = 3.3) in Social, 12.6 (SD = 2.9) in Spirituality, and 13.8 (SD = 2.8) in Environment. In all ART periods, HRQOL domain scores were significantly higher in HIV/AIDS patients without AUD than those with AUD, except Morbidity. Compared to other patients groups, patients who were in the 1st year of ART reported lower HRQOL, especially in the Physical domain (Figure 1).

**Table 1:** Alcohol use, adherence and health-related quality of life during ART

	**All**		**Duration of ART**									
			**Not-yet**		**<=1 year**		**1; <=2 year**		**2; <=4 year**		**4; <=7 year**	
**1. AUD**	**Mean**	**SD**	**Mean**	**SD**	**Mean**	**SD**	**Mean**	**SD**	**Mean**	**SD**	**Mean**	**SD**
AUDIT-C Score	2.0	2.3	2.4	2.5	2.1	2.4	2.1	2.3	1.8	2.1	2.0	2.5
	**N**	**%**	**N**	**%**	**N**	**%**	**N**	**%**	**N**	**%**	**N**	**%**
At-risk drinking	306	30.1	46	40.4	70	35.7	43	29.9	67	24.8	80	27.4
Binge drinking	227	22.3	28	24.6	40	20.4	33	22.9	65	24.1	61	20.9
**2. HRQOL Domain scores**	**AUD**		**Mean**	**SD**	**Mean**	**SD**	**Mean**	**SD**	**Mean**	**SD**	**Mean**	**SD**
Performance	No		12.8***	2.2	12.3**	2.5	12.8**	2.1	13.2***	2.3	13.0***	2.2
	Yes		11.6	1.9	11.5	2.4	12.0	2.1	12.2	2.3	12.2	2.1
Physical	No		13.8**	2.8	12.7**	3.3	13.9**	2.8	13.8**	2.7	13.7***	3.0
	Yes		12.5	3.4	11.8	3.2	12.8	3.3	12.9	3.7	12.5	2.9
Morbidity	No		12.5	3.9	12.3	3.3	12.6	3.1	12.9	3.7	13.2*	3.6
	Yes		12.5	3.7	12.2	3.4	12.9	3.3	13.1	3.5	12.6	3.5
Social	No		12.0***	2.9	11.0*	3.4	11.6**	3.4	11.6***	3.4	11.4*	3.3
	Yes		10.3	3.2	10.3	3.1	10.3	3.4	10.4	3.2	10.8	3.2
Spirituality	No		12.9***	2.9	12.5***	2.9	13.1***	2.8	13.1**	2.7	13.2***	2.8
	Yes		11.3	2.9	11.2	2.7	11.7	2.9	12.1	3.5	11.9	2.6
Environment	No		14.2***	2.5	13.6***	2.8	14.7***	2.5	14.4***	2.7	14.2***	2.6
	Yes		12.6	3.3	12.4	2.8	12.7	2.9	13.0	3.4	13.2	2.9
**3. ART Adherence**	**AUD**				**Mean**	**SD**	**Mean**	**SD**	**Mean**	**SD**	**Mean**	**SD**
**Mean VAS score**	No				94.9	8.5	95.3***	7.7	94.9	7.9	96.6***	4.8
	Yes				94.1	8.5	90.3	11.6	93.8	8.0	92.1	10.3
**% Non-adherence**					**N**	**%**	**N**	**%**	**N**	**%**	**N**	**%**
	No				23	21.3	18	19.2***	48	24.0	34	16.4***
	Yes				17	28.3	19	44.2	19	28.4	26	32.5

***p < 0.01, **p < 0.05, *p < 0.1 (Student-t test were used for comparing 2 means. Chi square tests were used for comparing 2 proportions).

### Associations of AUD with antiretroviral treatment adherence and HRQOL

Table 2 presents the association of AUD with ART adherence and HRQOL in multivariate analysis. Adjusting for propensity scores of AUD, there were small to medium decrements in five HRQOL domains scores (all except Morbidity) in patients who were hazardous drinkers, ranging from 0.3 (Social) to 0.5 (Environment). Compared to non-DU hazardous drinkers, at-risk drinkers who were also drug users reported a larger decrement in Environment, but a smaller decrement in Spirituality.

**Table 2:** Propensity score-adjusted differences in HRQOL and OR of non-adherence with regard to AUD in HIV/AIDS patients

	**All**			**Drug users (n = 468)**			**Non- drug users (n = 548)**		
	**Coef.**	**95% CI**	**Effect size**	**Coef.**	**95% CI**	**Effect size**	**Coef.**	**95% CI**	**Effect size**
**1. HRQOL outcomes**									
**At-risk drinking vs. None**									
Performance	-0.92***	(-1.24; -0.61)	-0.40	-0.80***	(-1.24; -0.36)	-0.35	-1.07***	(-1.52; -0.61)	-0.46
Physical	-0.97***	(-1.40; -0.54)	-0.31	-0.98***	(-1.55; -0.40)	-0.33	-0.97***	(-1.60; -0.33)	-0.31
Morbidity	-0.08	(-0.58; 0.41)	-0.02	0.09	(-0.60; 0.79)	-0.03	-0.26	(-0.97; 0.44)	-0.07
Social	-0.99***	(-1.45; -0.52)	-0.30	-1.04***	(-1.68; -0.39)	-0.31	-0.97***	(-1.64; -0.29)	-0.29
Spirituality	-1.34***	(-1.74; -0.94)	-0.46	-1.04***	(-1.56; -0.52)	-0.38	-1.68***	(-2.29; -1.07)	-0.55
Environment	-1.41***	(-1.81; -1.02)	-0.50	-1.70***	(-2.24; -1.16)	-0.60	-1.14***	(-1.71; -0.56)	-0.40
**Binge drinking vs. None**									
Performance	-0.04	(-0.40; 0.33)	-0.02	0.16	(-0.32; 0.65)	0.07	-0.25	(-0.82; 0.31)	-0.11
Physical	0.26	(-0.23; 0.76)	0.08	0.25	(-0.39; 0.89)	0.08	0.45	(-0.34; 1.25)	0.14
Morbidity	0.32	(-0.26; 0.89)	0.09	0.39	(-0.37; 1.14)	0.11	0.16	(-0.73; 1.06)	0.05
Social	0.66**	(0.12; 1.20)	0.20	0.48	(-0.23; 1.19)	0.15	0.88**	(0.04; 1.72)	0.26
Spirituality	0.11	(-0.36; 0.58)	0.04	0.16	(-0.40; 0.73)	0.06	-0.02	(-0.80; 0.76)	-0.01
Environment	0.23	(-0.24; 0.69)	0.08	0.11	(-0.51; 0.73)	0.04	0.46	(-0.27; 1.18)	0.16
	**OR**	**95% CI**		**OR**	**95% CI**		**OR**	**95% CI**	
**2. ART non-adherence**									
**At-risk drinking vs. None**	2.06***	(1.48; 2.85)		1.86***	(1.18; 2.95)		2.28***	(1.43; 3.62)	
**Binge drinking vs. None**	1.97***	(1.37; 2.82)		1.57*	(0.96; 2.56)		2.69***	(1.55; 4.67)	

***p < 0.01, **p < 0.05, *p < 0.1.

Binge drinking predicted HRQOL differently than at-risk drinking. HIV/AIDS patients who had binge drinking behaviour reported better HRQOL in five dimensions: Physical, Morbidity, Social, Spirituality, and Environment. However, the difference was small and statistically significant in only the Social domain.

During ART, patients who had AUD (both at-risk and binge drinking) were about twice as likely to be treatment non-adherent as those who did not have AUD. The odds ratio of non-adherence was higher in non-drug users than drug users.

## Discussions

This study found that AUD occurs with a high prevalence in large injection-driven HIV/AIDS epidemics in Vietnam, and supports the existing body of evidence of AUD’s negative effect on adherence to and outcomes of ART in such epidemics [36]. Moreover, this study contributes to the understanding of AUD’s influences on HRQOL outcomes of ART among HIV/AIDS patients. The magnitude of difference in HRQOL between patients with and without AUD in this study was comparable to a similar assessment in drug users [37]. Here, non-drug users were found to be even more likely than drug users to be non-adherent while engaging in either at-risk or binge drinking. Both hazardous and binge drinking problems strongly predicted non-adherence to ART; however their associations with dimensions of HRQOL were inconsistent. In different ART periods, hazardous drinking seemed to decrease, while binge drinking remained constant across all periods. Hazardous drinking was associated with small-to-medium decrements in almost all HRQOL dimensions, except Morbidity. Meanwhile, binge drinking was only associated with a small increase in the Social dimension.

This finding that binge drinking remained constant throughout the stages of ART and is associated with improved HRQOL in the Social dimension could be explained by the fact that alcohol use is legally accepted and culturally encouraged in Vietnamese society. Even though patients may perceive improved physical and mental health status during stable periods of ART, they may still have an AUD due to their continued binge drinking behaviour. In the modified WHOQOL-HIV BREF, the Social domain comprises 4 items, namely, social inclusion, financial resources, opportunities for acquiring new information and skills, and opportunities for recreation and leisure activities. Besides the slight increase among binge drinkers, social functioning was generally the poorest among the six HRQOL dimensions, particularly in at-risk drinkers. This may be explained by the fact that in the concentrated epidemic of Vietnam, the majority of patients have complex social backgrounds - including illicit drug use and sex work - that are accompanied by stigma and discrimination [38]. In addition, many HIV/AIDS patients do not have stable jobs, which prevent them from pursuing opportunities to live positively in their fight against HIV/AIDS.

This study’s findings suggest important implications for the HIV/AIDS intervention strategy in Vietnam. First, the strong association of AUD with poor adherence to and outcomes of antiretroviral treatment for HIV/AIDS patients highlights the necessity of screenings and interventions for AUD during ART. Given that the prevalence of AUD was high in drug users and non-drug users, such screening and intervention measures should be applied to all patient groups. Second, since adherence is central to achieving viral suppression and preventing drug resistance, intervention for ART adherence should be maintained throughout the stages of ART, and especially during the stable periods, beginning in the second year of treatment. While scaling up ART has substantially relieved the burden of HIV/AIDS in the country, we have found that benefits of ART might be limited if other interventions addressing the social and structural barriers associated with HIV/AIDS are not in place. Interventions that improve such aspects of HIV/AIDS patients’ lives as spirituality and social functioning might actually hold much potential to support ART adherence and outcomes. Finally, the findings inspire future studies to examine the underlying mechanisms of AUD and ART adherence and outcomes given the sociocultural and epidemiological characteristics of Vietnam.

The strengths of this study included a large sample size across different levels of the health system in 3 epicentres of Vietnam. In addition, we employed validated instruments which ensured improved psychometric properties and comparability of measurements. However, the study has some limitations that should be acknowledged. First, the cross-sectional design may not have allowed for the evaluation of the temporal relationships between AUD, patient adherence to ART, and HRQOL, and was limited in its ability to describe the changes during ART. In addition, the AUDIT-C questions referred to the patient’s lifetime drinking experience, thus, might not completely reflect the current behaviour. Self-reported alcohol use and ART adherence was also subject to biases due to patients’ recall or influences of health workers. However, comparing to other AUD measures, the AUDIT-C showed very good measurement properties in many studies, including some in Vietnamese populations [26,39-41]. In addition, the VAS for measuring ART adherence had been previously validated in the Vietnamese context, and showed convergent validity with the Adult AIDS Clinical Trials Group instrument [42].

## Conclusions

This study assessed the impact of AUD on ART adherence and HRQOL in HIV/AIDS patients in large injection-driven HIV epidemics in Vietnam. The magnitude of decrements in HRQOL outcomes suggests that screening and intervening for AUD is needed during ART, particularly during stable periods. Such social and psychological interventions may be extremely important to enhance patients’ responses to and outcomes of ART in Vietnam.

## Competing interests

The authors declare that they have no competing interests.

## Authors’ contributions

BXT and LTN designed the study and implemeted the survey. BXT analyzed the data. BXT, LTN, CDD, QLN, RMM wrote the manuscript. All authors read and approved the final manuscript.

## Pre-publication history

The pre-publication history for this paper can be accessed here:

http://www.biomedcentral.com/1471-2458/14/27/prepub
